# The effect of obstructive sleep apnea syndrome on serum S100B and NSE levels: a systematic review and meta-analysis of observational studies

**DOI:** 10.1186/s12890-020-1063-8

**Published:** 2020-02-05

**Authors:** Farzad Rezaei, Hooshyar Abbasi, Masoud Sadeghi, Mohammad Moslem Imani

**Affiliations:** 1 Department of Oral and Maxillofacial Surgery, Kermanshah University of Medical Sciences, Kermanshah, Iran; 2 Medical Biology Research Center, Kermanshah University of Medical Sciences, Kermanshah, Iran; 3 Students Research Committee, Kermanshah University of Medical Sciences, Kermanshah, Iran; 4 Department of Orthodontics, School of Dentistry, Kermanshah University of Medical Sciences, Kermanshah, Iran

**Keywords:** Obstructive sleep apnea, Brain, Serum, S100b, NSE

## Abstract

**Background:**

Obstructive sleep apnea syndrome (OSAS) is a common disorder that is accompanied by structural brain changes. This meta-analysis aimed to evaluate the effect of OSAS on the serum levels of astrocytic protein (S100B) and neuron-specific enolase (NSE) in observational studies.

**Methods:**

A comprehensive search was performed in the PubMed/Medline, Web of Science, Scopus, ScienceDirect, and Cochrane Library databases to assess the serum level of S100B and/or NSE in patients with OSAS and/or controls. The quality of the study was evaluated by the Newcastle-Ottawa Scale (NOS). A random-effects model was performed using RevMan 5.3 with the mean difference (MD) and 95% confidence intervals (CIs).

**Results:**

Out of 63 studies found in the mentioned databases and one identified by a manual search, nine studies were included and analyzed in this meta-analysis (three cross-sectional and six case-control studies). The analysis showed that the S100B [MD = 53.58 pg/ml, 95%CI: 1.81, 105.35; *P* = 0.04] and NSE levels [MD = 3.78 ng/ml, 95%CI: 2.07, 5.48; *P* < 0.0001] were significantly higher in patients than the controls. However, there were no significant differences between the S100B [MD = -28.00 pg/ml, 95%CI: − 79.48, 23.47; *P* = 0.29] and NSE levels [MD = 0.49 ng/ml, 95%CI: − 0.82, 1.80; *P* = 0.46].

**Conclusions:**

This meta-analysis found elevated serum S100B and NSE levels in OSAS patients compared to the controls, which suggests that these markers may be used as peripheral indicators of brain damage in OSAS.

## Background

Obstructive sleep apnea syndrome (OSAS) is a common disease that manifests as repeated events of nighttime breathing cessation because of upper airway collapse [1]. Epidemiological studies have shown that OSAS has a high incidence in the general population, with a prevalence of 2–4% [2]. There is a correlation between OSAS and increased platelet adhesiveness, vascular endothelial dysfunction and early symptoms of atherosclerosis, indicating an increased risk of vascular effects, such as stroke, in OSAS patients [3]. OSAS is accompanied by structural brain changes, and while how the brain is changed in OSAS remains unclear [4], OSAS likely causes brain injury [5]. Serum neuron-specific enolase (NSE) and astrocytic protein (S100B) concentrations have been examined under both clinical and experimental conditions to explain the relationship between neural cells and astrocytes in pathological situations [6, 7]. An elevated NSE level indicates neuronal injury, whereas an elevated S100B level may reflect either glial injury or astrocytic responses to neural damage [8]. These markers could serve as sensitive indicators of brain injury development [9]. The appearance of a biochemical marker of cerebral injury could serve as a considerable advantage in OSAS for identifying even small brain injuries and improving the efficacy of treatment [10].

The purpose of the present meta-analysis was to explore the effect of OSAS on serum S100B and NSE concentrations in observational studies.

## Methods

This meta-analysis was done based on the guidelines for the Preferred Reporting Items for Systematic Reviews and Meta-Analyses (PRISMA) [11].

### Search strategies

A comprehensive search was performed using the search terms “sleep apnea syndrome or apnea syndrome or sleep apnea or obstructive sleep or apnea syndrome or obstructive sleep apnea” and “S100B or astrocytic protein or NSE or neuron-specific enolase” in the PubMed/Medline, Web of Science, Scopus, ScienceDirect, and Cochrane Library databases without language restriction.

### Study selection

Three authors were involved in the selection of studies. The first author (M.S.) searched the studies, and the second author (M.M.I.) was blinded to the findings of the first reviewer. Any disagreements between the two authors were resolved by the third author (F.R.). All articles in this study were examined for an evaluation of the serum level of S100B and/or NSE in patients with OSAS and/or controls. The studies included in this meta-analysis met the following inclusion criteria: a) case-control or cross-sectional design; b) human study; and c) included the serum level of S100B and/or NSE. The exclusion criteria were as follows: a) duplication of a previous publication; b) review or case-series; c) conference paper; d) no full text; and e) no relevant data.

### Data extraction

Two authors (M.S & F.R) checked the studies included in meta-analysis and extracted the relevant data. We extracted the author name, publication year, country, patient number, mean age, percentage of males, body mass index (BMI), apnea-hypopnea index (AHI), type of method, and method features in each group.

### Quality assessment

The quality of the study was evaluated by the Newcastle-Ottawa Scale (NOS) [12]. One author (M.S) checked the quality of the studies. (The maximum total score was nine for case-control and cross-sectional studies. A high-quality study was considered a study with ≥7 stars. The quality of each study was evaluated by two authors (M.S. and M.M.I.) who reached a consensus via discussion.

### Statistical analyses

A random-effects model was used for analysis in Review Manager 5.3 (RevMan 5.3, The Cochrane Collaboration, Oxford, United Kingdom) using the mean difference (MD) and 95% confidence intervals (CIs). The heterogeneity between estimations was calculated by the Q and I^2^ statistics. For the Q statistic, heterogeneity was considered for *P* < 0.1. We graphically assessed publication bias using funnel plots and quantitatively evaluated bias using Begg’s test and Egger’s test in Comprehensive Meta-Analysis 2.0 as well as sensitivity analysis for evaluating the stability of the results using two strategies, the “cumulative analysis and one-study-removed”. *P* < 0.05 (two-sided) was considered statistically significant*.* The Wilcoxon test was used to compare the means among the OSAS severity grades. The units for S100B and NSE were pg/ml and ng/ml, respectively. In some studies, we estimated the “median (quartile),” [13], “median (range)” [14] or “mean (±SD).” The pooled mean and SD were obtained by the (N1*M1 + N2*M2/N1 + N2) and ((N1–1)*SD1 + (N2–1)*SD2/N1 + N2–2) formulas described in “http://crtha.iums.ac.ir/files/crtha/files/cochrane.pdf*.*”

## Results

**Fig. 1:**
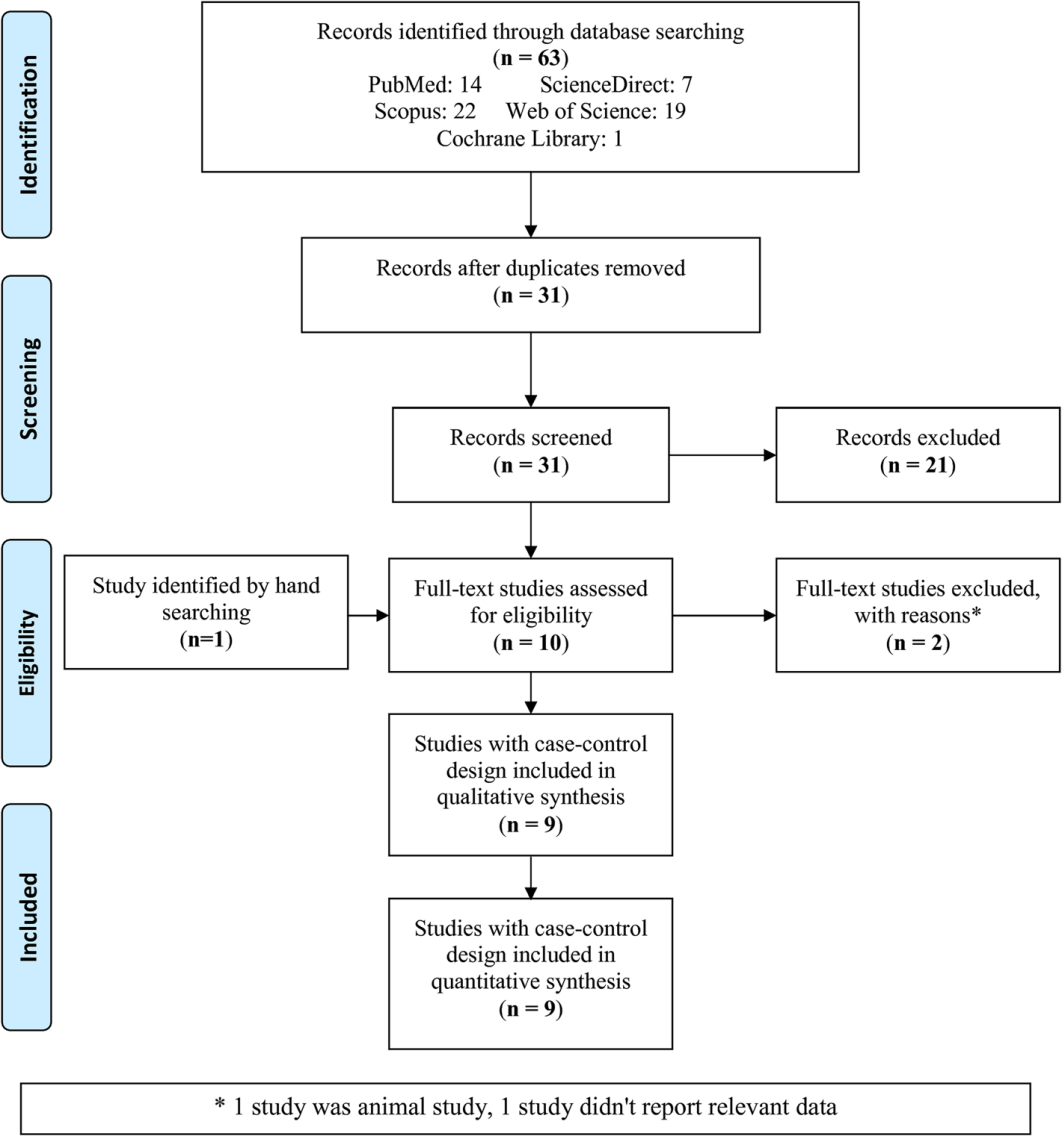
Flowchart of the literature search and study selection

A total of 63 studies were found in five databases. After removing duplicated studies, 31 articles were screened (Fig. 1). Out of the 31 studies screened, 21 articles were not relevant and therefore were excluded. Ten studies were evaluated for eligibility and one study identified by a manual search was added. Therefore, 11 studies were screened, 2 of which were excluded (one was an animal study and one did not report the relevant data). Finally, nine studies were included and analyzed in this meta-analysis.

Some characteristics of the studies included in the meta-analysis are shown in Table 1. The studies were published from 2002 to 2017. Two studies were reported in Germany [10, 21], two in Brazil [15, 17], one in the Czech Republic [16], three in Turkey [18–20], and one in Egypt [22]. Three were cross-sectional studies [10, 16, 17], and six were case-control studies [15, 18–22]. Other information is shown in Table 1.

**Table 1:** Characteristics of the studies included in meta-analysis (*n* = 9)

First author, year	Country	Type of study	Case (Number/^a^Mean age/^c^Male)	Control (Number/ ^a^Mean age/^c^Male)	Case (^b^BMI/ ^d^AHI means)	Control (^b^BMI/ ^d^AHI means)	Method		Method features	
							S100B	NSE	S100B	NSE
Jordan, 2002 [10]	Germany	CS	19/50.6/100	NA	32.8/−	NA	–	Cobas Core NSE enzyme immunoassay (EIA) kit of Hoffmann-La Roche AG (Basel, Switzerland)	–	The detection limit of this assay was 0.02 μg/L
Braga, 2006 [15]	Brazil	CC	29/38/100	17/35/100	25.9/27	26.9/2	Immunoluminescent assay kit (LIA-mat Sangtec 100 BYK-Sangtec, Germany)	ECLIA kit (Roche Diagnostics Corporation, USA)	Standard curve was linear up to 20 mg/L, and the coefficient variation was within 5%.	The coefficient of variation was less than 5%
Sonka, 2007 [16]	Czech Republic	CS	60/51.7/100	NA	NA	NA	ECLIA kit (Roche Diagnostics Corporation, USA)	–	–	–
da Silva, 2008 [17]	Brazil	CS	25/39.92/40	NA	47.86/36	NA	Immunoluminescent assay kit (LIA-mat Sangtec 100 BYK-Sangtec, Germany)	Elecsys-2010 (Roche Diagnostics Corporation®)	Standard curve was linear up to 20 mg/L, and the coefficient of variation was within 5%.	The coefficient of variation was less than 5%
Ergün, 2010 [18]	Turkey	CC	37/47/73	30/42/76.7	28.4/37	28.2/1	–	Radioimmunassay	–	Nor mal range was considered as 5–14 μmol/L
Duru, 2012 [19]	Turkey	CC	43/47.2/58.1	25/43.7/68	29.6/37.5	27/37.4	ELISA kit (Dia Metra, Italy)	–	–	–
Oztürk, 2012 [20]	Turkey	CC	26/56/80.7	28/56/71.4	28.8/51	27.7/−	ELISA kit (Bio Vendor Research and Diagnostic Products, Czech Republic)	–	Intra-assay and the inter-assay variation coefficients were 3.8 and 5.2%, respectively. Assay range was 0.05–2 μg/L	–
Traxdorf, 2016 [21]	Germany	CC	34/46.5/91.2	20/30.5/75	27.2/24.2	22.5/−	ELISA kit (Human S100B ELISA plate, Millipore, Darmstadt, Germany)	–	Cut-off limit of 0.10 μg/L (The approximate range of the system was 2.7–2000 pg/mL with a CV of 3% in intra-assay and 2–4.4% in the inter-assay analysis.)	–
Riad, 2017 [22]	Egypt	CC	55/44.1/56.4	34/45.1/50	35.7/12	30.6/2	ELISA kit (R&D Systems, Minneapolis, Minnesota)	–	The area under the curve was 0.998 with 96.4 sensitivity and 99.7% specificity at cutoff value of 21	–

*Abbreviations: CC* case-control, *CS* cross-sectional, *BMI* body mass index, *AHI* apnea–hypopnea index, *NSE* Neuron-specific enolase, *S100B* Astrocytic protein, *ECLIA* Electrochemiluminescent assay, *ELISA* Enzyme-linked immunosorbent *assay*

Units: ^a^year, ^b^kg/m^2^, ^c^ percent, and ^d^events/hour

### Quality assessment

Study-specific quality scores are summarized in Table 2. The mean score of the six case-control studies was 7.8. Four of them were awarded ≥7 stars. The mean score of the three cross-sectional studies was 7; three studies were awarded ≥7 stars and were defined as high-quality studies.

**Table 2:** Quality ratings for the studies included on the basis of Newcastle-Ottawa quality assessment scale (*n* = 9)

First author, year	Selection	Comparability	Outcome	Total score
Braga, 2006 [15]	3	1	2	6
Ergün, 2010 [18]	3	2	2	7
Duru, 2012 [19]	3	2	2	7
Oztürk, 2012 [20]	4	2	2	8
Traxdorf, 2016 [21]	3	1	2	6
Riad, 2017 [22]	3	2	2	7
Mean score (case-control studies)				7.8
Jordan, 2002 [10]	3	2	2	7
Sonka, 2007 [16]	3	2	2	7
da Silva, 2008 [17]	3	2	2	7
Mean score (cross-sectional studies)				7

**Fig. 2:**
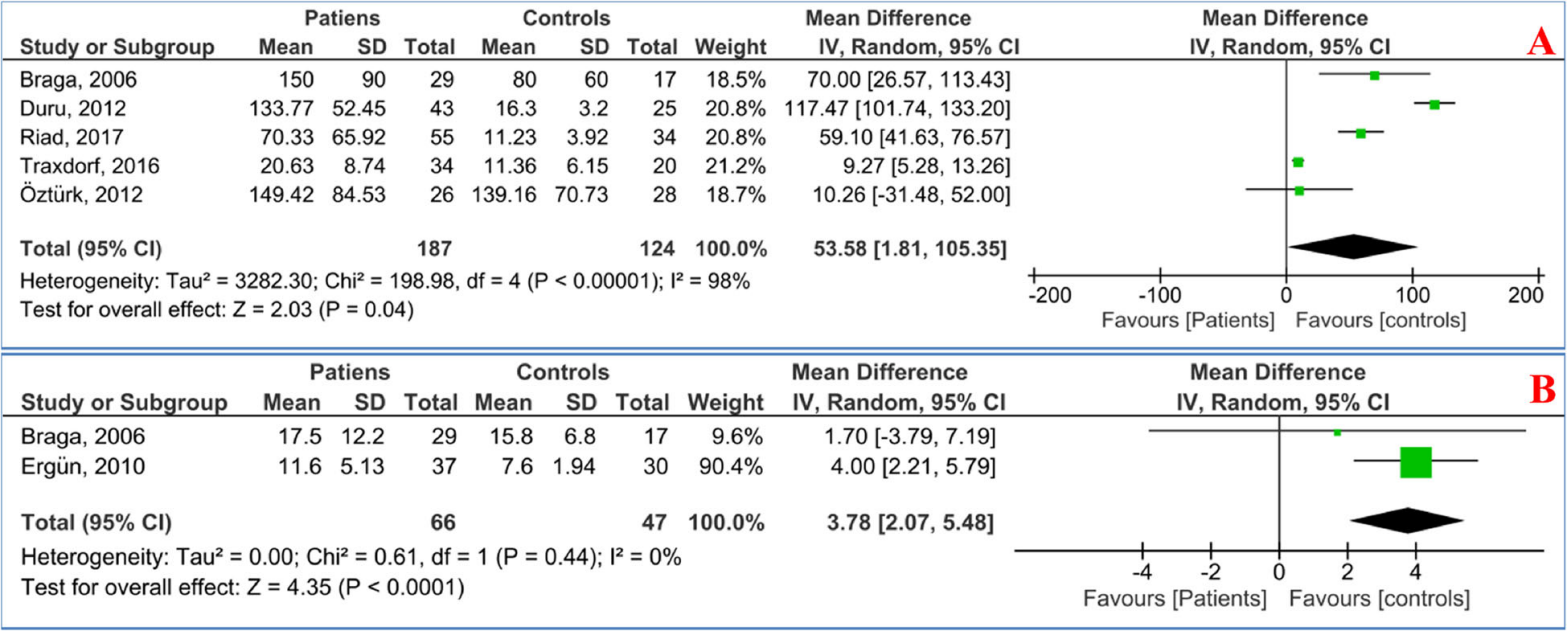
Forest plot of the random effects of the serum **a** S100B and **b** NSE levels in patients with OSAS compared to controls

Figure 2 shows the pooled MD of the serum S100B and NSE levels in OSAS patients compared to controls. The analysis shows that the S100B [MD = 53.58 pg/ml, 95%CI: 1.81, 105.35; *P* = 0.04, *I*^2^ = 98% (*P* < 0.00001)] and NSE levels [MD = 3.78 ng/ml, 95%CI: 2.07, 5.48; *P* < 0.0001, *I*^2^ = 0% (*P* = 0.44)] were significantly higher in the patients than in the controls.

**Fig. 3:**
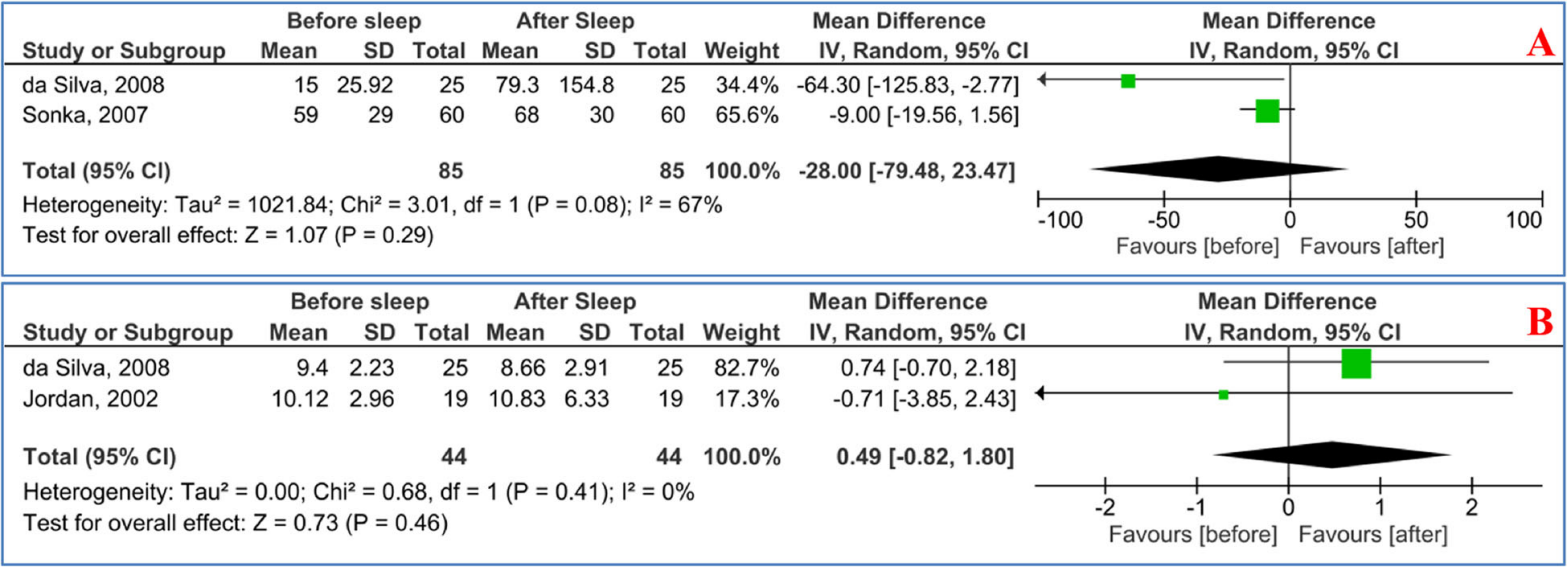
Forest plot of the random effects of the serum **a** S100B and **b** NSE levels in patients with OSAS before and after sleep

The pooled MD of the serum S100B and NSE levels in OSAS patients before and after sleep is shown in Fig. 3. There were no significant differences in the S100B [MD = -28.00 pg/ml, 95%CI: − 79.48, 23.47; *P* = 0.29, *I*^2^ = 67% (*P* = 0.08)] or NSE level [MD = 0.49 ng/ml, 95%CI: − 0.82, 1.80; *P* = 0.46, *I*^2^ = 0% (*P* = 0.41)].

Three studies [19, 21, 22] divided the severity of OSAS into three groups based on the AHI (mild, moderate, and severe). A comparison of the mean S100B level among these groups is shown in Table 3; no significant differences were found (*P* > 0.05).

**Table 3:** Comparison of serum S100B levels and severity of obstructive sleep apnea syndrome

First author, year	Mild (*n* = 13)	Moderate (*n* = 21)	Severe (*n* = 9)
Duru, 2012 [19]	126.67 ± 55.97	134.32 ± 61.55^a^	138.79 ± 58.83^ab^
	Mild (*n* = 5)	Moderate (*n* = 15)	Severe (*n* = 14)
Traxdorf, 2016 [21]	16.9 ± 5.26	21.5 ± 7.18^a^	20.37 ± 12.15^ab^
	Mild (*n* = 32)	Moderate (n = 14)	Severe (*n* = 7)
Riad, 2017 [22]	67.66 ± 53.3	60.5 ± 68.51^a^	91 ± 96.5^ab^

^a^ Wilcoxon test: *P* > 0.05 compared to mild. ^b^ Wilcoxon test: *P* > 0.05 compared to moderate

### Publication bias

**Fig. 4:**
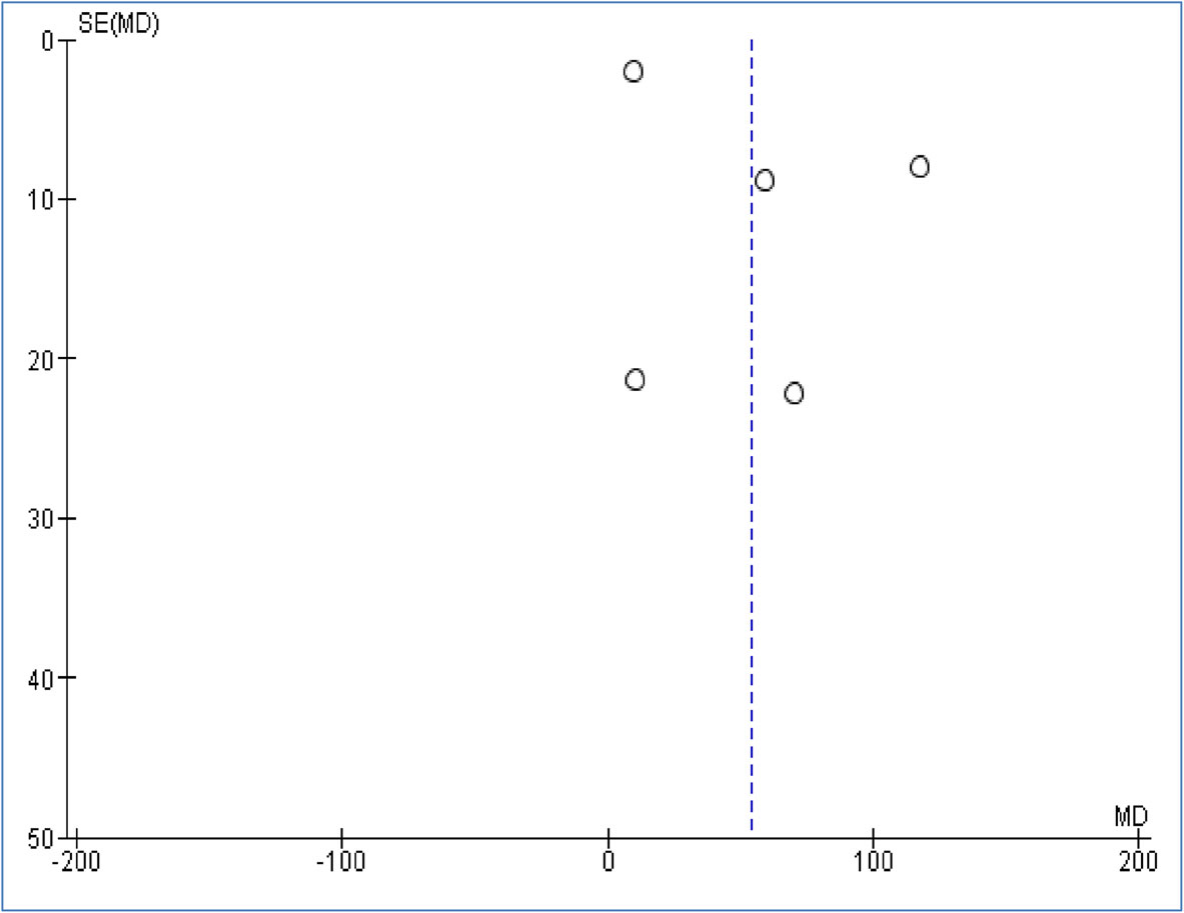
Funnel plot of the random effects of the serum S100B level in patients with OSAS compared to controls

Among the subgroup analysis, only one subgroup included more than two studies, allowing us to measure publication bias (S100B level in the patients compared to the controls) (Fig. 4). The points indicating the individual studies have a symmetric funnel plot and are distributed about the mean effect across the spectrum of precision levels and therefore Begg’s and Egger’s tests did not reveal the significant evidence of publication bias across the included studies.

### Sensitivity analysis

Both strategies- one study excluded” and “cumulative analysis”- were performed on an analysis with minimum three studies (S100B level in the patients compared to the controls) and did not qualitatively change the pooled OR. Therefore, these strategies revealed that the pooled OR was stable.

## Discussion

The meta-analysis evaluated serum S100B and NSE markers related to brain damages in OSAS patients. It was observed that serum levels of these markers in the patients were significantly higher than the controls, but the level changes were no significant after compared to before sleep.

OSAS can be related to cardiovascular and metabolic disorders, high blood pressure, diabetes, obesity, coronary artery illness [23, 24], stroke, and heart failure [25, 26]. OSAS can also cause cellular injury in the central nervous system (CNS) [27]. This meta-analysis shows that the serum S100B and NSE concentrations were significantly elevated in OSAS patients compared with the controls but that no significant changes occurred in the serum S100B and NSE levels of the patients during sleep. In addition, the S100B level was not significantly correlated with OSAS severity. Several studies [15, 19–22] reported higher serum S100B levels in OSAS patients than in the controls, indicating a significant difference in four studies [15, 19, 21, 22]. Two studies [15, 18] showed that the serum NSE level was significantly in OSAS patients higher than in the controls. The study by da Silva et al. [17] showed a significant difference in the serum S100B level after sleep compared with before sleep in the patients, but this difference was not confirmed by Sonka et al. [16]. In addition, two studies [10, 17] showed no significant difference in the serum NSE level after sleep compared to before sleep in the patients. In three studies [19, 21, 22] that assessed the S100B level in OSAS patients based on severity grade, there was no significant correlation between the S100B level and OSAS severity.

Studies of the *cerebrospinal fluid* (CSF) levels of S100B and NSE in patients with neurological injuries have shown a quantitative correlation between the degree of cell damage in the CNS and the level of these markers in CSF [28]. Clinically, elevated levels of NSE and S100B protein have been observed early following ischemic stroke, and elevated levels of S100B protein have been observed after intracerebral and subarachnoid hemorrhage [29]. The serum S100B level may increase not only due to glial injury but merely by opening the blood-brain barrier [30]. Cerebrovascular and neuropsychiatric disorders [31, 32], trauma, and stroke [33] can cause increased serum S100B protein levels due to S100B release from astrocytes.

In patients with mild and severe OSAS without neurological signs or a history of cerebrovascular events, there were no elevations in the serum NSE or S100B level [10]. da Silva et al. [17] reported a correlation between depression and elevated S100B levels in unhealthy obese patients. In addition, older patients are more likely to experience brain injury due to OSAS than younger patients [16]. It also appears that serum S100B levels are not dependent on age or sex [19, 34]. Duru et al. [19] reported no significant relationship between the serum S100B level and related factors of OSAS, including the AHI and patient characteristics, such as age and BMI. However, Sonka et al. [16] showed that the serum S100B concentration was inversely related to the AHI and directly related to both the basal and mean minimal oxygen saturation (SaO2) in line to the results of Riad’s study [22]. It has been confirmed that low mean nighttime SpO_2_ significantly associated with an elevated risk of a central nervous system events [35]. In OSAS patients, there is main effect of BMI on S100B level [22]. Duru et al. [19] reported that serum S100B levels did not correlate with age and BMI. Another study [36] showed that there is a negative correlation between serum S100B level and age in patients below 20 years, but after 20 years, S100B level has not appear to vary with age. Braga et al. [15] found a mild effect of age on S100B level, whereas Riad et al. [22] didn’t find any correlation between them. The studies checking SaO2 in OSAS patients compared to the controls showed that S100B [15, 16] and NSE [15] levels had no significant correlation with minimum SaO2 levels. Another study [17] showed a negative correlation between S100B levels and minimum SaO2 levels. Despite a few reported studies, S100B or NSE levels can be correlated with age, sex, AHI, and SaO2 levels. The studies reported different assay methods for S100B and NSE that some methods had a poor inter-assay that it can lead to inconsistent results [21] and reduce the reliability of results of the studies included in the meta-analysis. Therefore, it needs more studies in the future to confirm the effect of S100B or NSE levels on OSAS using unit methods for increasing the accuracy and reliability of the results.

The limitations of the study included the few studies in each analysis and differences among the studies in terms of age, sex, BMI, AHI, and different methods (different cut-offs or inter-assay).

## Conclusions

This meta-analysis found elevated serum S100B and NSE levels in OSAS patients compared to controls, which suggests that these markers could be used as peripheral indicators of brain *damage* in OSAS. Regarding the low number of the studies, the pooled analysis showed no significant correlation between the S100B level and OSAS severity or a difference in the S100B or NSE level after sleep compared to before sleep in the patients. Therefore, more case-control or cross-sectional studies are needed with an emphasis on demographic factors to confirm the results of this meta-analysis.

## Data Availability

The datasets used and/or analysed during the current study available from the corresponding author on reasonable request.

## Abbreviations

AHI: Apnea-hypopnea index
BMI: Body mass index
CI: Confidence interval
MD: Mean difference
NOS: Newcastle-Ottawa Scale
NSE: Neuron-specific enolase
OSAS: Obstructive sleep apnea syndrome
S100B: Astrocytic protein
